# Dietary Restriction and Neuroinflammation: A Potential Mechanistic Link

**DOI:** 10.3390/ijms20030464

**Published:** 2019-01-22

**Authors:** Eugene Bok, Myungjin Jo, Shinrye Lee, Bo-Ram Lee, Jaekwang Kim, Hyung-Jun Kim

**Affiliations:** Department of Neural Development and Disease, Korea Brain Research Institute (KBRI), Daegu 41062, Korea; eugenebok@kbri.re.kr (E.B.); jomj@kbri.re.kr (M.J.); srlee@kbri.re.kr (S.L.); leeboram@kbri.re.kr (B.-R.L.)

**Keywords:** dietary restriction, aging, neuroinflammation, astrocytes, microglia

## Abstract

Chronic neuroinflammation is a common feature of the aged brain, and its association with the major neurodegenerative changes involved in cognitive impairment and motor dysfunction is well established. One of the most potent antiaging interventions tested so far is dietary restriction (DR), which extends the lifespan in various organisms. Microglia and astrocytes are two major types of glial cells involved in the regulation of neuroinflammation. Accumulating evidence suggests that the age-related proinflammatory activation of astrocytes and microglia is attenuated under DR. However, the molecular mechanisms underlying DR-mediated regulation of neuroinflammation are not well understood. Here, we review the current understanding of the effects of DR on neuroinflammation and suggest an underlying mechanistic link between DR and neuroinflammation that may provide novel insights into the role of DR in aging and age-associated brain disorders.

## 1. Introduction

Aging is a naturally occurring multifactorial process that leads to morbidity and mortality. In particular, brain aging manifests as a gradual decline in memory and cognitive, executive, and motor functions. It is now widely accepted that brain aging is accompanied by region-dependent morphological and functional alterations. These include anatomical atrophy, reduction in brain volume, synaptic deficits, decline in the capacity for neurogenesis, cytoskeletal abnormalities, mitochondrial dysfunction, an increase in reactive oxygen species (ROS) and oxidized proteins, a reduction in neurotrophic factors, impairment of the blood–brain barrier (BBB), and induction of chronic neuroinflammation (reviewed in [1,2]). 

In experimental and clinical settings, dietary restriction (DR) is induced by reducing the caloric intake and/or intermittent fasting. Since McCay and colleagues first reported the effect of DR on lifespan in rats in 1935 [3], DR has been shown to be the most robust and reliable experimental intervention for extending longevity. Accumulating evidence suggests that DR extends lifespan in an evolutionary scale from worms to rodents [4]. Although the effects of DR on longevity and brain function in nonhuman primates and human beings are still controversial [5,6,7,8,9,10,11,12], a recent report from the CALERIE (Comprehensive Assessment of Long-term Effects of Reducing Intake of Energy) trial noted that two-year-long DR in healthy, nonobese human subjects caused weight loss and reduction in energy expenditure and oxidative stress [13]. These findings indicate that the mechanism underlying DR-mediated antiaging may be evolutionally conserved from worms to mammals, possibly including humans.

A growing body of evidence demonstrates that DR exerts its beneficial effects on brain aging at multiple levels. Although there is some degree of discrepancy across studies, likely due to the difference in the model organisms and experimental design, DR appears to mitigate all of the morphological and functional alterations in the brain associated with aging (reviewed in [1,14,15,16,17]). 

A major hallmark of aging is systemic, low-grade chronic inflammation throughout the body, termed inflamm-aging, a term coined by Claudio Franceschi in 2000 [18]. Notably, these inflammatory signs are similar to the ones associated with obesity and metabolic diseases [19,20], providing a possible glimpse into why DR exerts anti-inflammatory effects on aging-associated inflammation. As with other organs, chronic low-grade inflammation is a common feature of the aged brain [21]. Neuroinflammation is a host defense mechanism against harmful stimuli and damage in the brain. However, chronic inflammation can be deleterious in normal aging as well as in pathological aging related to neurodegenerative diseases. The central nervous system (CNS) is composed of heterogeneous cell types, including neurons, microglia, astrocytes, and oligodendrocytes. Although two major glial cell types, astrocytes and microglia, are known to be key players in inflammatory responses in the brain, it is now well recognized that all neural cells participate to some degree in the neuroinflammatory responses. Neuroinflammation often manifests as astrogliosis, microgliosis, and an increase in secreted inflammatory mediators, such as cytokines, chemokines, and complement proteins. Accumulating evidence from clinical and basic research suggests that neuroinflammation is tightly connected to the decline in brain function during aging [22]. In this article, we review the evidence that DR has an anti-neuroinflammatory effect and suggest an underlying mechanistic link between DR and neuroinflammation that may provide novel insights into the role of DR in aging and age-associated brain disorders. 

## 2. Neuroinflammation in the Aged Brain

### 2.1. Evidence of Increased Neuroinflammation with Age

The brain was once considered to be an absolute immune-privileged organ isolated from the systemic immune system by the BBB. In fact, the brain is an immunologically active organ that communicates with the immune and endocrine systems. Moreover, circulating cytokines and chemokines can signal to the CNS, although the underlying mechanisms are still elusive [23,24]. Similar to the changes seen in the systemic immune system with aging, numerous studies have revealed that the inflammatory status of the brain increases during normal aging. The hallmarks of brain aging include impairment of DNA repair, accumulation of oxidative damage, and inflammatory activation of glial cells [21]. Consistent with this, induction of the genes associated with immune and inflammatory responses is observed in most human brain regions during normal aging, although the changes vary across brain regions [25,26,27]. Moreover, proinflammatory cytokines, such as interleukin-1 beta (IL-1β), tumor necrosis factor alpha (TNF-α), and interleukin-6 (IL-6), are increased in multiple brain regions, including hippocampus and cortex, during normal aging [28,29,30,31,32,33,34]. Furthermore, some immune regulatory molecules and anti-inflammatory cytokines, including interleukin-10 (IL-10) and interleukin-4 (IL-4), were found to be reduced in aged rodent brains [35,36]. In addition, the aged brain is more vulnerable to peripheral systemic inflammatory stimuli [37]. Hypothalamic inflammation impairs the brain-governed energy control and glucose homeostasis, thereby promoting peripheral inflammation as well as neuroinflammation [14].

Another hallmark of neuroinflammation is gliosis. Gliosis is the focal proliferation and activation of glial cells (astrocytes, microglia, and oligodendrocytes) in the CNS in response to various insults. Overwhelming evidence shows that astrogliosis is increased in multiple brain regions during normal aging in both rodents and humans [38,39,40]. Activation of astrocytes is observed relatively early in adult brains during aging, as evidenced by increased expression of glial fibrillary acidic protein (GFAP) [40,41,42,43,44,45,46]. Signs of microgliosis and microglial activation, such as induction of major histocompatibility complex II (MHC II), scavenger receptor (CD86), and CD40, are also prominent in the aged brain [37,41,42,47,48,49,50]. Microglia isolated from the aged mouse brains were found to be more sensitive to inflammatory stimuli than those from the young brains [37].

Taken together, these studies demonstrate that even healthy aged brains have a significant increase in neuroinflammatory responses, as evidenced by increased gliosis, elevated levels of inflammatory cytokines, and decreased levels of anti-inflammatory molecules. 

### 2.2. Microglia in the Aged Brain

Microglia are the innate immune cells in the CNS that play a pivotal role in maintaining microenvironment homeostasis, synaptic plasticity, and immune surveillance [51,52,53]. During embryogenesis, microglial progenitor cells originated from yolk sac-derived myeloid migrate into the CNS before the BBB construction is completed [54,55]. Once the BBB construction is completed, the peripheral hematopoietic system does not further contribute to the microglia pool of the CNS under normal condition, meaning that the adult microglia population is maintained by self-renewal in the brain [56,57]. In addition, microglia are long-lived cells with the median lifetime of 15 months in mouse neocortex, and only about 26% of microglia are replaced per year [58]. Réu and colleagues reported that microglia in the human brain is on average 4.2 years old, and the median renewal rate is 28% per year [59]. A long life span of microglia may make them more susceptible to the aging-related changes compared to other neural cells. 

Microglia play a key role in neuroinflammation associated with aging. Microglia in the young adult brain, which are typically in a quiescent resting state, become activated in response to various types of insults. With aging, microglia develop an altered phenotype compatible with proinflammatory activation. The elevated inflammatory profile of microglia during aging is closely associated with a “primed” state. “Primed” microglia have a higher basal expression of inflammatory response genes, a lower threshold for inflammatory activation, and elevated reactivity following an immune challenge [60]. Many inflammatory markers, such as MHC II, complement receptor 3 (CD11b), Toll-like receptors (TLRs), CD86, CD11b, and CD11c, are upregulated in microglia of the aged brain [61,62,63]. Among TLRs, TLR2–4 are known to be important for the ATP-dependent secretion of IL-1β in primed microglia [64]. Besides molecular changes, morphological changes in microglia also occur in the aged brain from rodent to human. Microglia in the aged brain show a “de-ramified” morphology characterized by short processes and reduced dendritic branching, suggesting an activation of microglia with age [65,66,67,68,69]. 

Godbout and colleagues showed that peripheral injection of lipopolysaccharide (LPS), a powerful inducer of the inflammatory response, caused an exaggerated and prolonged neuroinflammatory response in the aged brain [29]. The elevated inflammatory responses in aged mice were associated with sustained behavioral deficits, such as reduced motor function and altered social behavior [29]. In addition, elevated levels of IL-1β after systemic injection of LPS were mainly derived from MHC II (a marker for primed microglia)-positive microglia in the aged mouse brain [37]. Consistent with this, systemic injection of *Escherichia coli* resulted in an exaggerated and prolonged upregulation of IL-1β in hippocampus of aged rats compared with young adults [70]. Aged rodents and older individuals showed enhanced neuroinflammation, increased neurodegeneration, and poorer recovery after traumatic brain injury (TBI) than their younger counterparts [71,72,73]. Aged mice experienced more severe neuronal damage upon TBI induction by controlled cortical impact that young mice [72]. Moreover, MHC II was strongly upregulated in microglia of the aged TBI brain [72]. Taken together, these reports indicate that primed microglia play an important role in enhancing neuroinflammatory responses to immune challenges in the aged brain.

The effect of aging on microglia gene expression was recently investigated through transcriptome analysis in microglia isolated from young and aged mouse brains [74]. Consistent with the characteristics of aged microglia, genes associated with the immune, phagosome, lysosome, oxidative phosphorylation, and antigen presentation signaling pathways were significantly affected by aging [74]. It is noteworthy that the transcriptional profile of aged microglia was clearly different from that of M1 macrophage, M2 macrophages, or acutely activated microglia [74]. A list of differentially expressed genes (DEG) between young and aged microglia associated with the immune, inflammatory responses, and antigen presentation signaling pathways is summarized in Table 1 [74]. 

### 2.3. Astrocytes in the Aged Brain

Astrocytes are the most abundant cell type in the mammalian brain. Astrocytes are essential for neuroprotection against excitotoxicity, ROS, insults, and extracellular overload of potassium ions [75]. They also have functions associated with synaptic plasticity and trophic support for neurons [75]. Similar to microglia, astrocytes display an elevated inflammatory profile with age, including morphological and molecular alterations. For example, astrocytes in young human subjects were found to have long and slender processes, whereas astrocytes in aged brains possessed short and stubby processes [76]. In addition, upregulation of GFAP and vimentin has been reported in astrocytes of aged brains [60]. Notably, increased expression of GFAP and vimentin is a typical signature of reactive astrocytes [77,78]. Thus, these findings indicate that astrocytes become reactive with age. 

Upon immune challenge to the CNS, such as with an injury, activated astrocytes secrete various inflammatory mediators, such as chemokines, cytokines, and growth factors [79]. Astrocytes interact with microglia to regulate inflammatory responses in the brain. For instance, orosomucoid-2 (ORM2) derived from astrocytes effectively inhibited the proinflammatory activation of microglia via C-C chemokine ligand 4 (CCL4) during the late phase of neuroinflammation [80]. Recently, Liddelow and colleagues reported that activated microglia can induce the formation of A1 reactive astrocytes, a neurotoxic inflammatory astrocyte [81], by secreting cytokines, including IL-1α, TNFα, and C1q [77]. A subset of genes associated with reactive astrocytes was upregulated in the aged brain of wild-type mice, whereas their upregulation was significantly attenuated in mice lacking *Il-1α*, *Tnfα*, and *C1q* [82]. These data suggest that Il-1α, TNFα, and C1q are critical for activation of astrocytes in the aged brain. 

Recently, two groups performed transcriptomic analyses in astrocytes isolated from multiple regions of young and aged mouse brains [82,83]. Both studies suggest that astrocytes have region-specific transcriptional identities and that their transcriptional changes with age are also region-dependent. Moreover, compared with young astrocytes, aged astrocytes show a stronger gene expression profile associated with reactive astrocytes [82,83]. A list of aging-induced DEG in astrocytes associated with immune responses, inflammatory responses, and antigen presentation signaling pathways is summarized in Table 2.

## 3. The Effects of Dietary Restriction on Neuroinflammation

### 3.1. The Effects of Dietary Restriction on Neuroinflammation in Normal Aging

The beneficial effects of DR on cognition and memory are under debate, with some studies reporting beneficial effects and others showing no benefits in the aging process [1,16,84,85,86,87,88,89,90,91,92,93]. However, there is agreement across studies that DR exerts anti-inflammatory effects against aging-driven neuroinflammation [15,17,94,95]. DR attenuated aging-driven increase in GFAP levels in multiple brain regions, including hypothalamus [96,97], hippocampus [97,98], corpus callosum [41], and cortex [97] in middle-aged rodents. Short-term DR in middle age in rhesus macaques also attenuated astrogliosis in hippocampus, suggesting conservation of the effects of DR on astrogliosis from rodents to nonhuman primates [5]. Downregulation of GFAP levels by DR is regulated at least in part at the transcriptional level [38,41]. Rozovsky and colleagues reported that neurite outgrowth was significantly reduced in cortical neurons cultured together with astrocytes derived from 24-month-old rats compared with those from 3-month-old rats [99]. Interestingly, knock-down of GFAP by RNAi diminished effects of aged astrocytes on neurite outgrowth, suggesting that the role of activated astrocytes in normal aging is not restricted to inflammatory regulation in the brain. Besides astrogliosis, DR also attenuated aging-driven microgliosis in corpus callosum, striatum, hippocampus, and hypothalamus in rodents [32,38,41,100]. DR downregulated circulating inflammatory mediators in the periphery [32,101,102]. In line with this, short-term DR in old age attenuated aging-associated induction of inflammatory cytokines, such as IL-1β in mouse hippocampus [28], TNF-α, and IL-6 in rat hypothalamus [32]. 

Morgan and colleagues showed that activation of astrocytes and microglia in rat brain was regulated by chronic DR from young to middle age in a region-specific manner [41]. Besides inflammation, DR also regulated aging-associated decrease in Sirtuin 1 (SIRT1) [103,104] and Brain-derived neurotrophic factor (BDNF) [105,106] in the brain in a region-specific manner, raising the possibility that different regulatory pathways may respond to DR or that the same regulatory pathways may respond to DR to different extents in different brain regions. Of note, gross effects of DR seen in the aged brain were not observed at young age [96,107,108], suggesting that molecular pathways regulated by DR are somehow inert in the brain at young age or that they respond to DR only when they are dysregulated over certain thresholds with advancing age. 

Several studies have performed transcriptome analyses in dietary-restricted rodents during aging [109,110,111,112]. A number of genes associated with inflammation or the immune response showed changed expression levels under DR in aged rodent brains. When the DEGs for aged microglia were compared with the immune-related DEGs that were affected by DR, genes involved in antigen processing and presentation via MHC II were found in both lists (Table 1 and Table 3, *H2-eb1, Ctse, H2-aa, H2-ab1,* and *Cd74*). As mentioned above, MHC II is a marker for primed microglia. Interestingly, recent transcriptome and proteome analyses of human aged microglia have shown that genes and proteins related to antigen processing and presentation are significantly affected by aging [113]. Furthermore, overall age-related increases in the genes associated with antigen processing and presentation via MHC I have been reported in astrocytes (Table 2, [114]). Therefore, it is possible that the antigen processing and presentation pathway is a possible mechanistic link between DR and neuroinflammation.

### 3.2. The Effects of Dietary Restriction in Age-Related Neurodegenerative Diseases

Neuroinflammation is a major pathological hallmark of many neurodegenerative diseases, such as Alzheimer’s disease (AD), Parkinson’s disease (PD), amyotrophic lateral sclerosis (ALS), and multiple sclerosis (MS) [115,116,117,118]. So far, several studies have evaluated the beneficial effects of DR on AD in different mouse and monkey models. AD is pathologically characterized with abnormal accumulation of amyloid plaques and neurofibrillary tangles (NTFs), mainly composed of extracellular amyloid beta (Aβ) and intracellular tau, respectively [119]. Short-term DR before amyloid plaque accumulation significantly decreased accumulation of amyloid plaques and GFAP levels in two different mouse models of amyloidosis, J20 (DR for 6 weeks) and Tg2576 (DR for 14 weeks) [120]. J20 line expresses human *APPswe/ind* with the *K670N/M671L* (Swedish) and the *V717F* (Indiana) mutations [121], whereas Tg2576 line expresses human *APPswe* mutant with *PS1* with *M146L* mutation [122]. Chronic DR for 12 months similarly decreased amyloid plaque burden in the brain of Tg25765 mice [123,124]. In another study, Mouton and colleagues sought to evaluate the therapeutic potential of DR at advanced pathologic stage in *APPswe/PS1ΔE9* mouse model of AD [125]. *APPswe/PS1ΔE9* mice aged 13–14 months with severe accumulation of amyloid plaques were subjected to DR for 4.5 months. DR significantly reduced amyloid plaque deposition in the brain, suggesting that DR may represent a novel therapeutic strategy for patients with advanced AD. It is noteworthy that DR attenuated amyloid pathology only in female Tg2576 and not in male mice [123]. By contrast, DR attenuated amyloid deposition in male *APPswe/PS1ΔE9* line [125]. This conflicting finding may be due to different mouse models, different experimental settings, and/or different DR regimen in those two studies. Further investigations are needed to fully address whether sex influences the neuroprotective effects of DR on AD. Wu and colleagues demonstrated that DR attenuated astrogliosis in a double knockout of *Psen1* and *Psen2*, another AD mouse model [126]. Ghrelin agonist, which induces hunger, also reduced the levels of insoluble Aβ and microgliosis in hippocampus of *APPSwDI* mice [127]. Whether DR reduces amyloid deposition in nonhuman primates is debatable. Although life-long DR reduced Aβ levels in temporal cortices of Squirrel monkeys [128], short-term DR in middle age did not reduce amyloid plaque burden in aged rhesus macaques [5]. However, DR attenuated astrogliosis in hippocampal CA region and entorhinal cortex in aged rhesus macaques [5]. In mouse models of tau pathology, DR has generated inconsistent results. Chronic DR reduced both Aβ and phospho-tau levels in hippocampus in the triple transgenic mouse model of AD, 3xTg mice expressing human *APP KM670/671NL, TAU P301L*, and *PSEN1 M146V* mutants with concomitant improvement in memory [129]. In a mouse model only expressing human mutant Tau (Tg4510), DR partially rescued memory deficits without altering tau accumulation, neuronal loss, or the levels of astroglial (GFAP) and microglial activation (Iba-1) [130]. 

PD is characterized by selective loss of dopaminergic neurons in the substantia nigra region [131]. DR ameliorated the loss of dopaminergic neuron and motor deficits in a 1-methyl-4-phenyl-1,2,3,6-tetrahydropyridine (MPTP)-induced moue model of PD [132]. By contrast, no beneficial effects of DR were observed in 6-hydroxydopamine-induced rat model of PD [133]. In a MPTP-induced PD model of rhesus monkey, six months of DR improved locomotor activity and increased the levels of dopamine and its metabolites in striatum [134]. It is notable that DR attenuated MPTP-induced loss of dopaminergic neuron, astrogliosis, and microgliosis in substantia nigra in WT but not ghrelin KO mice, suggesting that ghrelin mediates the neuroprotective effects of DR in MPTP-induced mouse model of PD [135]. DR also attenuated pathologic events in a mouse model of Huntington’s disease (HD) [136]. 

In animal models of ALS, DR has generated conflicting results in different studies. Chronic DR accelerated clinical onset and progression and shortened the lifespan while transiently improving motor performance in a mouse model of ALS expressing human *SOD1 G93A* mutant [137], possibly by increasing lipid peroxidation, inflammation, and apoptosis [138]. In another study with the same model, no benefit of DR on disease onset or progression was observed [139]. However, DR significantly delayed the onset of disease and extended the lifespan in a different mouse model of ALS expressing *SOD1 H46R/H48Q* mutant [140]. Because of the substantial discrepancies across studies, it is difficult to draw a firm conclusion as to whether DR is beneficial in experimental models of ALS. 

MS is a chronic inflammatory neurodegenerative disorder characterized by demyelination in the CNS [141]. In an experimental allergic encephalomyelitis (EAE), a rodent model of MS, 15 days of severe DR (66% food restriction) before EAE induction in 6 week-old male rats significantly attenuated progression of EAE [142]. In contrast to severe calorie restriction (66% food restriction), 15 days of mild DR (33% food restriction) before EAE induction in 6-week-old male rats had no inhibitory effect on the development of acute EAE [143]. Five weeks of mild DR (40% food restriction) before EAE induction in 5-week-old mice ameliorated clinical EAE with less severe inflammation, demyelination, and axon injury [144], suggesting that the effects of DR on EAE are dependent on severity and duration of regimen. A recent study demonstrated that periodic cycles of fasting mimicking diet with very-low-calorie and low-protein lasting 3 days every 7 days ameliorated demyelination and promoted oligodendrocyte precursor cell regeneration in a mouse model of EAE [145]. Although there is a report suggesting the beneficial effect of saturated fat restriction on MS in humans [146], it is currently not clear whether DR has protective and/or therapeutic effect on MS in humans.

## 4. A Potential Mechanistic Link between Dietary Restriction and Neuroinflammation

Although the precise mechanisms of DR’s neuroprotective functions are not fully elucidated, it has been suggested that DR exerts neuroprotective effects through multiple pathways, such as modulating metabolic rates, reducing oxidative stress, increasing anti-inflammatory responses, regulating insulin sensitivity, and improving synaptic plasticity and neurogenesis (reviewed in [15,16]). All of the molecular changes induced by DR may directly or indirectly contribute to the regulation of neuroinflammation associated with aging and neurodegenerative diseases. DR may directly mitigate activation of glial cells and modulate expression of inflammatory cytokines and indirectly regulate neuroinflammation by reducing inflammatory stresses, such as accumulation of toxic proteins and oxidative stress. 

A previous gene profiling study provided evidence that DR increased IκBα, a NF-κB inhibitor, and decreased the p65 subunit of NF-κB in mouse neocortex [147]. Besides regulating expression of NF-κB, DR also reduced phosphorylation and activity of NF-κB in the brain of a mouse model of experimental astrocytoma [148]. These findings suggest that DR suppresses inflammation by inhibiting NF-κB signaling in the brain. It is well established that inflammation induces ROS generation in various cell types. In turn, ROS can activate redox-sensitive NF-κB, forming a positive feed-forward loop [149,150]. An increase of oxidative stress in the brain is a hallmark of aging as well as neurodegenerative diseases. It is evident that DR reduces oxidative stress in senescent astrocytes as well as in aged brains, as evidenced by reduction in ROS and protein oxidation [16,151,152,153,154]. Although how DR reduces oxidative stress in the brain remains elusive, several potential mechanisms of DR’s antioxidative functions have been proposed [155]. A recent transcriptome study showed that DR increased expression of ROS scavengers, such as glutathione S-transferases and thioredoxins, in cortices of rats [111]. In another study, Hyun and colleagues demonstrated that DR increased activities of multiple enzymes related to plasma membrane redox system and antioxidants, such as α-tocopherol and coenzyme Q10 [152]. DR also attenuated age-dependent induction of NADPH oxidase 2 (NOX2) in hypothalamus, which may contribute to the reduction of ROS by DR in the aged brain [32]. Taken together, it is likely that DR ameliorates neuroinflammation associated with aging and neurodegenerative diseases at least in part by reducing oxidative stress and thereby suppressing inflammatory responses in the brain.

Cellular redox status can also regulate SIRT1, a regulator of oxidative stress and inflammation [156]. SIRT1-mediated deacetylation of p65 subunit of NF-κB inhibits inflammatory responses via suppressing NF-κB signaling pathway [157]. SIRT1 can also regulate oxidative stress by modulating FOXO3, which regulates expression of antioxidant genes, such as MnSOD [158]. It is also notable that SIRT1 can regulate cellular redox status by modulating mitochondrial biogenesis by inducing PGC1-α and nitric oxide synthase [156,159]. Oxidative stress is known to suppress expression and activity of SIRT1 at the transcriptional and posttranslational levels [14,156]. In line with this, reduced SIRT1 expression has been reported in the brains of aged rodents [103,160]. Several lines of evidence have shown that SIRT1 level is increased by DR in multiple brain regions, including hypothalamus, hippocampus, and cortex [103,104,161,162]. Of note, it has been reported that *Sirt1* transgenic mice have phenotypes that resemble DR [163] and show better physical activity in response to DR than wild-type mice [104]. By contrast, *Sirt1*-deficient mice exhibit defects in somatotropic and behavioral responses to DR [104,164]. SIRT1, together with NF-κB, seem to lie at the hub of antioxidative and anti-inflammatory responses mediated by DR in the brain. 

Besides NF-κB and SIRT1-mediated pathways, several other pathways have been suggested as potential mechanisms mediating the anti-inflammatory action of DR, such as modulation of BBB permeability and regulation of steroid hormones in hypothalamic–pituitary–adrenal axis, such as glucocorticoid [20,165]. However, it is not clear whether these pathways indeed mediate anti-inflammatory action of DR in the brain due to a lack of solid evidence. Further studies are warranted to comprehensively understand mechanisms of anti-neuroinflammatory action of DR. 

## 5. Conclusions

**Figure 1:**
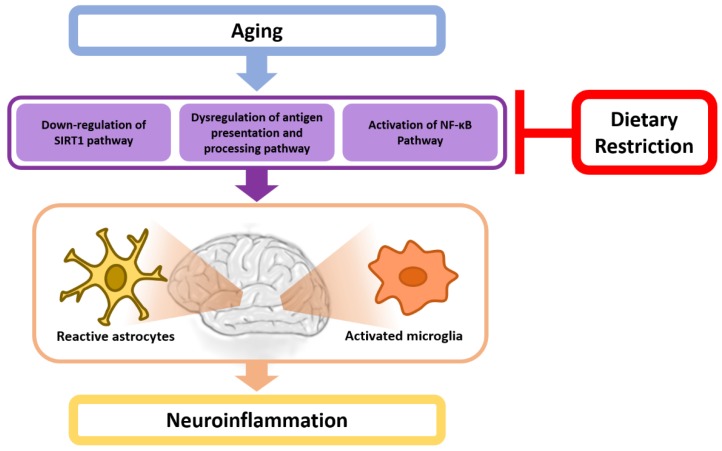
Overview of the potential mechanistic links between age-induced neuroinflammation and dietary restriction. All arrows indicate stimulation/consequences, and red T bar indicates mitigation.

There is overwhelming evidence that DR attenuates inflammatory responses associated with aging in the brain. Figure 1 shows the possible relationship between DR and neuroinflammation during aging. However, the molecular bases of DR-mediated anti-inflammatory responses in the brain remain elusive. Moreover, it is not clear whether DR is neuroprotective for age-related neurodegenerative diseases because of mixed results. Thus, further in-depth studies are warranted to fully elucidate the molecular mechanisms of anti-inflammatory responses mediated by DR and whether DR can represent a novel therapeutic intervention for neurodegenerative diseases.

## Figures and Tables

**Table 1:** List of differentially expressed genes (DEGs) associated with inflammation/immune response in aged microglia.

Gene Name	GO Term	Expression Level Old vs. Young	Age/Brain Region	Species	Reference
*Axl*	GO:0002376, GO:0045087, GO:0006954	Up	24 months/whole brain	*Mus musculus*	[74]
*Camp*	GO:0045087	Up	24 months/whole brain	*Mus musculus*	[74]
*Ccl3*	GO:0006954, GO:0050729, GO:0006955	Up	24 months/whole brain	*Mus musculus*	[74]
*Cd274*	GO:0006955	Up	24 months/whole brain	*Mus musculus*	[74]
*Cd36*	GO:0006955	Up	24 months/whole brain	*Mus musculus*	[74]
*Cd74*	GO:0002376, GO:0006955, GO:0019886, GO:0042613	Up	24 months/whole brain	*Mus musculus*	[74]
*Chst1*	GO:0006954	Up	24 months/whole brain	*Mus musculus*	[74]
*Clec7a*	GO:0045087, GO:0006954	Up	24 months/whole brain	*Mus musculus*	[74]
*Ctse*	GO:0019886	Up	24 months/whole brain	*Mus musculus*	[74]
*Cxcl13*	GO:0006954, GO:0006955	Up	24 months/whole brain	*Mus musculus*	[74]
*Cybb*	GO:0045087, GO:0006954	Up	24 months/whole brain	*Mus musculus*	[74]
*H2-aa*	GO:0002376, GO:0006955, GO:0019886, GO:0042613	Up	24 months/whole brain	*Mus musculus*	[74]
*H2-ab1*	GO:0002376, GO:0006955, GO:0019886, GO:0042613	Up	24 months/whole brain	*Mus musculus*	[74]
*H2-eb1*	GO:0002376, GO:0006955, GO:0019886, GO:0042613	Up	24 months/whole brain	*Mus musculus*	[74]
*Ifit3*	GO:0002376, GO:0045087	Up	24 months/whole brain	*Mus musculus*	[74]
*Ifitm2*	GO:0002376	Up	24 months/whole brain	*Mus musculus*	[74]
*Ifitm3*	GO:0002376, GO:0045087	Up	24 months/whole brain	*Mus musculus*	[74]
*Lcn2*	GO:0002376, GO:0045087	Up	24 months/whole brain	*Mus musculus*	[74]
*Lgals3*	GO:0002376, GO:0045087	Up	24 months/whole brain	*Mus musculus*	[74]
*Ltf*	GO:0002376	Up	24 months/whole brain	*Mus musculus*	[74]
*Ly9*	GO:0002376, GO:0045087	Up	24 months/whole brain	*Mus musculus*	[74]
*Oasl2*	GO:0002376, GO:0045087, GO:0006955	Up	24 months/whole brain	*Mus musculus*	[74]
*Rsad2*	GO:0002376, GO:0045087	Up	24 months/whole brain	*Mus musculus*	[74]
*S100a8*	GO:0002376, GO:0045087, GO:0006954, GO:0050729	Up	24 months/whole brain	*Mus musculus*	[74]
*S100a9*	GO:0002376, GO:0045087, GO:0006954, GO:0050729	Up	24 months/whole brain	*Mus musculus*	[74]
*Spp1*	GO:0006954	Up	24 months/whole brain	*Mus musculus*	[74]

GO:0002376, immune system process; GO:0006954, inflammatory response; GO:0006955, immune response; GO:0019886, antigen processing and presentation of exogenous peptide antigen via MHC class II; GO:0042613, MHC class II protein complex; GO:0045087, innate immune response; GO:0050729, positive regulation of inflammatory response. All gene listed passed the rule that log2 fold change >1 and adjusted *p* value <0.05.

**Table 2:** List of DEGs associated with inflammation/immune response in aged astrocytes.

Gene Name	GO Term	Expression Level Old vs. Young	Age/Brain Region	Species	Reference
*Akap8*	GO:0002376, GO:0045087	Up	24 months/Striatum	*Mus musculus*	[82]
*App*	GO:0045087	Up	24 months/Striatum	*Mus musculus*	[82]
*B2m*	GO:0006955, GO:0002376, GO:0045087	Up	24 months/ Hippocampus, Striatum	*Mus musculus*	[82]
*Bcl6*	GO:0002376, GO:0006954	Up	24 months/ Cortex, Striatum	*Mus musculus*	[82]
*Bmp6*	GO:0006954	Up	24 months/ Visual cortex, Striatum	*Mus musculus*	[82,83]
*Bst2*	GO:0045087, GO:0002376	Up	24 months/Motor cortex	*Mus musculus*	[83]
*C3*	GO:0045087, GO:0002376, GO:0006954	Up	24 months/Motor cortex, Visual cortex	*Mus musculus*	[83]
*C4b*	GO:0045087, GO:0006954	Up	24 months/Motor cortex, Visual cortex, Striatum	*Mus musculus*	[82,83]
*Csf1*	GO:0002376, GO:0045087, GO:0006954	Up	24 months/Striatum	*Mus musculus*	[82]
*Ctss*	GO:0019882, GO:0006955	Up	24 months/ Hippocampus, Striatum	*Mus musculus*	[82]
*Cxcl10*	GO:0006955, GO:0006954	Up	24 months/ Hippocampus, Striatum	*Mus musculus*	[82]
*Cxcl12*	GO:0006955	Down	24 months/Hippocampus	*Mus musculus*	[82]
*Cxcl5*	GO:0006954	Up	24 months/Visual cortex	*Mus musculus*	[83]
*Defb1*	GO:0045087	Up	24 months/Motor cortex, Visual cortex	*Mus musculus*	[83]
*Enpp2*	GO:0006955	Down	24 months/Hippocampus	*Mus musculus*	[82]
*Erap1*	GO:0002376	Up	24 months/Striatum	*Mus musculus*	[82]
*H2-d1*	GO:0019882, GO:0006955, GO:0002376	Up	24 months/Cortex, Hippocampus, Striatum	*Mus musculus*	[82]
*H2-k1*	GO:0019882, GO:0006955, GO:0002376	Up	24 months/ Hippocampus, Striatum	*Mus musculus*	[82]
*Hfe*	GO:0019882	Up	24 months/Striatum	*Mus musculus*	[82]
*Icosl*	GO:0002376	Up	24 months/Striatum	*Mus musculus*	[82]
*Ifit1*	GO:0045087, GO:0002376	Up	24 months/Visual cortex, Striatum	*Mus musculus*	[82,83]
*Ifit3*	GO:0002376, GO:0045087	Up	24 months/Cortex, Striatum	*Mus musculus*	[82]
*Ifitm3*	GO:0002376, GO:0045087	Up	24 months/Cortex, Striatum	*Mus musculus*	[82]
*Ly86*	GO:0002376, GO:0045087, GO:0006954	Up	24 months/Striatum	*Mus musculus*	[82]
*Nlrp6*	GO:0045087, GO:0002376, GO:0006954	Up	24 months/Visual cortex	*Mus musculus*	[83]
*Oasl2*	GO:0045087, GO:0002376, GO:0006955	Up	24 months/Motor cortex, Visual cortex, Hippocampus, Striatum	*Mus musculus*	[82,83]
*Psmb8*	GO:0002376, GO:0019882	Up	24 months/Visual cortex, Hippocampus, Striatum	*Mus musculus*	[82,83]
*Psmb9*	GO:0002376, GO:0019882	Up	24 months/ Hippocampus, Striatum	*Mus musculus*	[82]
*Rsad2*	GO:0045087, GO:0002376	Up	24 months/Visual cortex	*Mus musculus*	[83]
*Serinc3*	GO:0002376, GO:0045087	Up	24 months/Striatum	*Mus musculus*	[82]
*Serping1*	GO:0002376, GO:0045087	Up	24 months/Striatum	*Mus musculus*	[82]
*Tspan2*	GO:0006954	Up	24 months/Striatum	*Mus musculus*	[82]
*Tyrobp*	GO:0045087	Up	24 months/Striatum	*Mus musculus*	[82]
*Zc3hav1*	GO:0045087, GO:0002376	Up	24 months/Visual cortex, Striatum	*Mus musculus*	[82,83]

GO:0019882, antigen processing and presentation; GO:0002376, immune system process; GO:0006954, inflammatory response; GO:0006955, immune response; GO:0045087, innate immune response. All gene listed passed the rule that log2 fold change >1 and adjusted *p* value <0.05.

**Table 3:** List of DEGs associated with inflammation/immune response in dietary restriction during aging.

Gene Name	GO Term	Expression Level DR vs. AL	Age/Brain Region	Species	Reference
*Prlr*	GO:0034097	Down	15 months/Hippocampus	*Mus musculus*	[109]
*Il2ra*	GO:0034097	Up	15 months/Hippocampus	*Mus musculus*	[109]
*Sigirr*	GO:0034097	Down	15 months/Hippocampus	*Mus musculus*	[109]
*Ptk2b*	GO:0002376	Up	15 months/Hippocampus (CA1)	*Mus musculus*	[112]
*Bcl6*	GO:0006954, GO:0050727, GO:0002376	Up	15 months/Hippocampus (CA1)	*Mus musculus*	[112]
*Ccr1*	GO:0006954	Up	15 months/Hippocampus (CA1)	*Mus musculus*	[112]
*Il1r1*	GO:0050727	Up	15 months/Hippocampus (CA1)	*Mus musculus*	[112]
*Tnfrsf25*	GO:0006954	Up	15 months/Hippocampus (CA1)	*Mus musculus*	[112]
*Gal*	GO:0006954	Up	15 months/Hippocampus (CA1)	*Mus musculus*	[112]
*H2-q10*	GO:0002376	Up	15 months/Hippocampus (CA1)	*Mus musculus*	[112]
*S100a8*	GO:0006954, GO:0050727, GO:0002376	Up	15 months/Hippocampus (CA1)	*Mus musculus*	[112]
*S100a9*	GO:0006954, GO:0050727, GO:0002376	Up	15 months/Hippocampus (CA1)	*Mus musculus*	[112]
*C1qbp*	GO:0006955	Up	28 months/Cerebral cortex	*Rattus norvegicus*	[111]
*Rt1-db1*	GO:0006955, GO:0019886, GO:0042613, GO:0002504	Up	28 months/Cerebral cortex	*Rattus norvegicus*	[111]
*Rt1-ba*	GO:0006955, GO:0019886, GO:0042613, GO:0002504, GO:0019882	Up	28 months/Cerebral cortex	*Rattus norvegicus*	[111]
*Cxcl12*	GO:0006955	Up	28 months/Cerebral cortex	*Rattus norvegicus*	[111]
*Rt1-da*	GO:0006955, GO:0042613, GO:0002504, GO:0019882	Up	28 months/Cerebral cortex	*Rattus norvegicus*	[111]
*Cd74*	GO:0006955, GO:0019886, GO:0042613, GO:0019882	Up	28 months/Cerebral cortex	*Rattus norvegicus*	[111]
*Rt1-bb*	GO:0006955, GO:0019886, GO:0042613, GO:0002504, GO:0019882	Up	28 months/Cerebral cortex	*Rattus norvegicus*	[111]
*Fcer1g*	GO:0019886	Up	28 months/Cerebral cortex	*Rattus norvegicus*	[111]
*Rab3b*	GO:0019882	Up	28 months/Cerebral cortex	*Rattus norvegicus*	[111]
*Tnfaip6*	GO:0006954	Down	19 months/Hypothalamus	*Mus musculus*	[110]
*C1qg*	GO:0002376	Up	19 months/Hypothalamus	*Mus musculus*	[110]

GO:0002376, immune system process; GO:0002504, antigen processing and presentation of peptide or polysaccharide antigen via MHC class II; GO:0006954, inflammatory response; GO:0006955, immune response; GO:0019882, antigen processing and presentation; GO:0019886, antigen processing and presentation of exogenous peptide antigen via MHC class II; GO:0034097, response to cytokine; GO:0042613, MHC class II protein complex; GO:0050727, regulation of inflammatory response. All gene listed passed the rule that log2 fold change >0.5 and adjusted *p* value <0.05.

## Abbreviations

ROS	Reactive oxygen species
DR	Dietary restriction
CALERIE	Comprehensive Assessment of Long-term Effects of Reducing Intake of Energy
BBB	Blood–brain barrier
IL-1β	Interleukin-1 beta
TNF-α	Tumor necrosis factor alpha
IL-6	Interleukin-6
GFAP	Glial fibrillary acidic protein
MHC II	Major histocompatibility complex II
TLRs	Toll-like receptors
Iba-1	Ionized calcium-binding adapter molecule 1
LPS	Lipopolysaccharide
TBI	Traumatic brain injury
CCL4	C-C chemokine ligand 4
DEG	Differentially expressed genes
ORM2	Orosomucoid-2
BDNF	Brain-derived neurotrophic factor
SIRT1	Sirtuin 1
AD	Alzheimer’s disease
PD	Parkinson’s disease
ALS	Amyotrophic lateral sclerosis
NTFs	Neurofibrillary tangles
Aβ	Amyloid beta
MPTP	Methyl-4-phenyl-1,2,3,6-tetrahydropyridine
MS	Multiple sclerosis
HD	Huntington’s disease
AL	Ad libitum
EAE	Experimental allergic encephalomyelitis
NOX2	NADPH oxidase 2
